# Experimental validation and identification of ferroptosis-associated biomarkers for diagnostic and therapeutic targeting in hearing loss

**DOI:** 10.3389/fnagi.2025.1526519

**Published:** 2025-04-25

**Authors:** Chenyang Yuan, Tianyu Ma, Mengting Liu, Li Jiang, Gongrui Tang, Qi Hu, Tianhong Zhang

**Affiliations:** Department of Otorhinolaryngology Head and Neck Surgery, The First Affiliated Hospital of Harbin Medical University, Harbin, Heilongjiang, China

**Keywords:** hearing loss, ferroptosis, age-related hearing loss, biomarkers, aging

## Abstract

**Objectives:**

Ferroptosis, a regulated form of cell death, has attracted significant attention in hearing loss research; however, the role of ferroptosis-related genes remains unclear. This study aimed to clarify diagnostic and therapeutic targeting of ferroptosis-related genes in hearing loss.

**Methods:**

Differentially expressed genes related to hearing loss from the GEO database were intersected with ferroptosis-related genes. The Lasso and SVM-RFE models were applied to reduce the gene set, identifying model genes. Biological functions, pathways, and gene-drug associations related to these model genes were analyzed. Age-related hearing loss (ARHL) genes within the model genes were obtained from a genome-wide association study (GWAS) dataset. Further validation was conducted in HEI-OC1 cells and the cochleae of C57BL/6J mice, including auditory brainstem response (ABR) testing, qRT-PCR, Western blotting, Fe^2+^ detection, and immunofluorescence analysis.

**Results:**

The study identified 20 ferroptosis-related genes associated with hearing loss. Using Lasso and SVM-RFE models, a novel model was constructed, consisting of nine genes (*SCD, ENPP2, PANX2, NEDD4, MEF2C, ABCC5, KLHDC3, CYP4F8* and *IFNA2*). Among these, *MEF2C and NEDD4* were found to be associated with ARHL.

**Conclusion:**

Ferroptosis is a potential pathological mechanism in hearing loss research, and the nine ferroptosis-related genes identified provide promising targets for exploring new diagnostics and treatments for hearing loss. Notably, *MEF2C* and *NEDD4* are associated with ARHL.

## 1 Introduction

It is estimated that 2.5 billion individuals across the globe will be living with different forms of hearing loss by 2050 (Chadha et al., 2021; GBD 2019 Hearing Loss Collaborators, 2021). Various factors, including familial predisposition, aging, exposure to noise and medication use contribute to the risk of hearing loss (Vlajkovic and Thorne, 2021). Collectively, these factors exert a comprehensive influence on the progression of hearing loss, culminating in pathophysiological alterations to the inner ear. These modifications encompass damage of inner and outer hair cells, atrophy of the stria vascularis, basement membrane thickening, calcification, and hyalinization (Tawfik et al., 2020; Wu et al., 2020). Research indicates that programmed cell death pathways play an important role in the molecular mechanisms of hearing loss. Ferroptosis, a form of newly programmed cell death, is characterized by disruptions in iron metabolism, free radical accumulation, and lipid peroxidation, and has garnered increasing attention in this field (Sun et al., 2022). Ferroptosis involves intracellular iron overload, free radical accumulation, and lipid peroxidation (Dixon et al., 2012). Recently, ferroptosis has gained attentions in many research fields including degenerative diseases (Doll et al., 2019; Tang et al., 2021). Similarly, ferroptosis plays a pivotal role in HC injury, and its importance is evident even in the degeneration of the auditory cortex (Chen et al., 2020; Ma et al., 2022). However, there is limited research on the relationship between hearing loss, age-related hearing loss, and ferroptosis.

In recent years, lots of human non-syndromic hearing impairment loci have been mapped and some model genes identified. Some research has focused on genes and proteins that may play a role in the development of hearing loss, but the majority of genes and significant therapeutic targets remain unclear. This study aims to integrate databases and apply bioinformatics methods to analyze the involvement of ferroptosis-related genes and associated signaling pathways in the development of hearing loss. Moreover, we screen Genetic variation of model genes associated with age-related hearing loss (ARHL) and verify the expression of some important model genes in the cochlea. The goal is to provide new insights into the pathogenesis and treatment of hearing loss.

## 2 Materials and methods

### 2.1 Data source and patient selection

We acquired 734 datasets by querying the GEO database^1^ with the keywords “hearing loss,” which were eventually narrowed down to 24 datasets after applying the terms “Expression profiling by array” and “Homo sapiens.” Subsequently, GSE9822 (Bykhovskaya et al., 2009) was selected by eliminating datasets with inadequate sample sizes. The dataset chosen comprised of gene expression data and clinical data from 14 deaf individuals and 29 normal individuals. Ineligibility for inclusion in the study was ascribed to patients with incomplete survival data or an overall survival of less than 30 days. The chosen dataset consists of expression data from lymphoblastoid cell lines derived from peripheral blood lymphocytes from deaf patients and unaffected family members, all of whom carry the deafness-related mutation in the 12S rRNA gene, and unaffected unrelated controls who do not. The study utilized datasets that were readily accessible to the public and had already been granted ethical approval for the original research.

### 2.2 Data correction and extraction of ferroptosis genes expression

First, the gene probe matrix file was converted to a gene expression file. Then, the expression data of the control and experimental samples were read separately, and log2 processing was applied to the data for correction. We retrieved a total of 728 ferroptosis genes from the FerrDb database^2^, including driver genes, suppressor genes, and marker genes. We then extracted the expression levels of these ferroptosis genes by comparing them with the expression data files. Finally, we analyzed the differentially expressed genes. The study was conducted according to the principles expressed in the Declaration of Helsinki. All the datasets were collected from published literature, and written informed consent was confirmed.

### 2.3 LASSO model and SVM-REF model genes selection

Using the “glmnet” package, construct a Lasso regression model, plot the graph of Lasso regression and the graph of cross-validation, find the point with the minimum cross-validation error, and its value is the number of selected genes for output. A regression model is a statistical technique used to understand the relationship between one or more independent variables (predictors) and a dependent variable (outcome). And using the “e1071” package, we build a machine learning model based on the support vector machine recursive feature elimination algorithm (SVM-REF). This model will be used to rank the importance of genes and perform cross-validation to obtain accuracy and error metrics. Cross-validation is a method used to assess the predictive performance of a model by partitioning the data into subsets. The model is trained on some subsets while being tested on others, allowing for a more robust evaluation of its accuracy and generalizability. The output of the SVM-REF model will correspond to the number of genes that result in the highest cross-validation accuracy and the smallest error (Wu et al., 2024).

### 2.4 Functional and pathway enrichment analysis

We conducted a pathway and functional enrichment analysis of the Kyoto encyclopedia of genes and genomes (KEGG) (Kanehisa and Goto, 2000) and Gene Ontology (GO) (Ashburner et al., 2000) using various R packages, including “clusterProfiler,” “complexHeatmap,” “org.Hs.eg.db,” “DOSE,” “ggplot2,” “circlize,” “dplyr,” and “enrichplot.” We used the BH algorithm to correct for all *p*-values. To examine the enrichment of intersecting genes, we utilized the GSEA database for pathway and process analysis.

Gene Set Enrichment Analysis (GSEA) is a bioinformatics method used to analyze gene expression data. It helps researchers determine whether a set of genes is differentially expressed under specific conditions and whether these genes play an important role in biological processes or signaling pathways (Subramanian et al., 2005). GSVA (Gene Set Variation Analysis) is a tool used to analyze sets of genes, which can transform individual gene expression profile data into gene set expression profile data, thus better reflecting the biological features of gene sets. It is commonly used in the analysis of RNA sequencing data to identify differentially expressed gene sets and discover pathway enrichment of gene sets (Hänzelmann et al., 2013).

Gene Set Enrichment Analysis (GSEA) and GSVA (Gene Set Variation Analysis) are both methods used to assess the enrichment of predefined gene sets, but they differ in approach and application. GSEA compares two groups of samples (e.g., control vs. treatment) to evaluate gene set enrichment based on ranked gene expression, often using permutation testing to assess significance. It is suited for experiments with clear group labels. GSVA, on the other hand, provides enrichment scores for individual samples, assessing pathway activity without the need for predefined group labels. It is ideal for analyzing complex datasets with sample heterogeneity, such as cancer studies. GSVA uses a non-parametric approach to capture gene set variation across samples. While GSEA focuses on group comparisons, GSVA is more flexible for analyzing gene set variation at the single-sample level.

### 2.5 Analysis of immune cell infiltration

Cell-type Identification by Estimating Relative Subsets of RNA Transcripts (CIBERSORT) is a computational method used to estimate the composition of different cell types in tissue samples. It utilizes transcriptomic data to infer the relative proportions of various cell types within a sample, particularly in the context of mixed cell populations, such as in studies of the tumor microenvironment or immune cell populations. CIBERSORT uses a machine learning algorithm called Support Vector Regression (SVR) (Newman et al., 2015). In order to perform immune cell infiltration analysis on the corrected sample files, we utilized the “e1071” and “preprocessCore” packages. These packages are commonly used tools in the R language for pattern recognition and data preprocessing, enabling us to process and analyze the sample data to obtain valuable insights into immune cell infiltration.

### 2.6 Drug-gene interactions and associated RNA prediction

Drug-gene interactions refer to the effect of a drug on the activity of a gene, or the effect of a genetic variant on the response to a drug. These interactions are important in determining the effectiveness and safety of drug therapy. We downloaded drugs associated with the feature genes from the Dgidb database^3^ (Cannon et al., 2024) and generated network relationship and node attribute files. These files were visualized using Cytoscape software (3.9.2).

TargetScan^4^ (McGeary et al., 2019) was developed in 2003 by Benjamin Lewis and colleagues at MIT. It predicts miRNA target genes by searching for conserved sites that match the seed region of each miRNA. As an option, non-conserved sites can also be predicted. Unlike other target prediction tools, TargetScan provides an accurate ranking of predicted targets for each miRNA. miRanda^5^ (John et al., 2004) is a bioinformatics tool for miRNA target prediction, developed in 2003 by Anton Enright and colleagues at the Memorial Sloan-Kettering Cancer Center. Written in C, miRanda screens the 3′-UTR based on three main criteria: sequence matching, the thermodynamic stability of the miRNA-mRNA duplex, and the conservation of target sites. miRDB^6^ (Liu and Wang, 2019) uses a machine learning model trained on a large set of experimental data to predict interactions between miRNAs and target genes and to assign a Target Score. The score ranges from 0 to 100, with higher scores indicating higher reliability of the predicted interactions. spongeScan^7^ (Furió-Tarí et al., 2016) is able to identify putative miRNA binding patterns in lncRNA sequences. In the web tool, expression data can be added to the predicted representation, which greatly facilitates downstream functional analysis. SpongeScan is different from other lncRNA-miRNA interaction prediction websites that utilize CLIP-seq data, in that it allows for extensive searching of user-provided data and can be used for any organism with sequence information.

### 2.7 Screening genetic variations in model genes associated with age-related hearing loss

To validate whether model genes are involved in ARHL in human, we conducted in-depth analysis using the previously published GWAS dataset for age-related hearing impairment (ARHI) (GWAS CATALOG: GCST90012115). This GWAS dataset gathers large-scale genomic data to analyze the genetic background of age-related hearing impairment, also known as ARHL (Kalra et al., 2020). The summary statistics data were downloaded from the European Bioinformatics Institute (EBI)^8^. Genes of interest were screened for SNPs reaching nominal significance (*p* < 0.01) in their genomic loci.

### 2.8 HEI-OC1 cell culture

The HEI-OC1 cell line, provided by Dr. Iris Heredia of the School of Engineering at the University of California, Los Angeles (UCLA), originates from the House Ear Institute-Organ of Corti. These cells were maintained in high-glucose Dulbecco’s Modified Eagle Medium (DMEM) supplemented with 10% fetal bovine serum (FBS, Gibco, United States) and 1% ampicillin (Sangon Biotech, Shanghai). Cultivation was conducted under controlled conditions at 33°C with a 10% CO_2_ atmosphere. Subsequently, the HEI-OC1 cells underwent a 48 h treatment with 30 mg/ml D-galactose (D-gal) (Sigma, United States) to simulate an aging model.

### 2.9 Cell viability quantification and measurement of Fe^2+^

Cell counting Kit-8 (CCK8) (Dojindo, Japan) was used to examine cell viability according to manufacturer’s introduction. We used FerroOrange (Dojindo) to detect intracellular Fe^2+^ according to the manufacturer’s protocol. HEI-OC1 cells were treated with D-gal and D-gal+Fer-1 for the indicated amount of time and stained with a final concentration of 1 μmol/l FerroOrange. FerroOrange for 30 min at 37°C. Images were acquired using LSM 710 confocal microscope (Zeiss, Oberkochen, Germany). We selected eight randomly chosen regions from each group and measured the fluorescence intensity of Fe^2+^. Each experiment was repeated three times.

### 2.10 Real time PCR and western blotting

In order to verify the expression of model genes, quantitative real-time PCR was conducted. Following treatment with 30 mg/ml D-gal, total RNA was extracted from HEI-OC1 cells and cochleae using the EZ-press RNA Purification Kit (EZBioscience, United States). Following RNA extraction, reverse transcription into cDNA was conducted using the Monad kit (MR05201, Monad Biotech Co., Ltd., China), according to the manufacturer’s instructions. Subsequently, qPCR was performed using the Takara kit (RR420A, Japan) on a LightCycler 480 instrument (Roche, United States). The primer sequences and genes utilized are detailed in Table 1. GAPDH was employed as an internal normalization control. And we used the 2^–ΔΔCT^ method to analyze relative gene expression, which calculates fold changes in gene expression by normalizing target gene expression to an internal control and comparing it to a reference sample (Maren et al., 2023).

**TABLE 1:** Primers used for qRT-PCR.

Genes	Forward primer (5′ to 3′)	Reverse primer (5′ to 3′)
*Mef2c*	GTGGTTTCCGTAGC AACTCCTAC	GGCAGTGTTGAAGC CAGACAGA
*Nedd4*	TGCTTTTGCCTACT TCATCTGG	ATGTGGTGGTTTTAGA GTTGTGG
*Klhdc3*	CCGACTGCTTTTCC AACGACATC	CCAGCATTGTGGC TGAGTGGAA
*Abcc5*	GCAAACTGGTTGGAA TCTGCGG	CAAAGGTCCCACTGA CGGCAAT
*Gapdh*	CATCACTGCCACCCA GAAGACTG	ATGCCAGTGAGCTT CCCGTTCAG

The cochleae were lysed with RIPA lysis buffer (Beyotime, China) and centrifuged at 14,000 × *g* for 20 min at 4°C. Supernatants were separated and transferred to PVDF membranes (0.2 μm, Millipore, Bedford, MA, United States). The membranes were incubated overnight at 4°C with the primary antibodies: Mef2c and GAPDH (1:1000, Proteintech, China). Subsequently, the membranes were incubated with goat anti-rabbit IgG (1:3000, Proteintech) for 1 h. After detecting by ECL kit (Beyotime, China).

### 2.11 Animals and auditory brainstem response threshold test

The male C57BL/6J mice aged 28 days (*n* = 6) and 12 months (*n* = 6) were purchased from Shanghai Sipeifu Laboratory. All mice were housed in groups of six per cage with free access to food and water, and subjected to a 12 h light/dark cycle. All procedures were conducted in accordance with the “Guiding Principles in the Care and Use of Animals” (China) and were approved by the Institutional Animal Care and The Ethics Committee of First Hospital Affiliated to Harbin Medical University (protocol number IACUC-2023092). And all methods are reported in accordance with ARRIVE guidelines.

The ABR system was obtained from Tucker-Davis Technologies (Alachua, FL, United States). Mice were anesthetized via intraperitoneal injection of 1% pentobarbital sodium (70 mg/kg), and their body temperature was maintained at 37°C using a thermostatic heating pad. The recording electrode was placed subcutaneously at the vertex of the skull, while the reference and ground electrodes were positioned on either side of the mastoid. Hearing thresholds were assessed using six tone burst frequencies (4, 8, 16, 22.6, 32, and 45.2 kHz). The stimulus intensity of the tone bursts started at 90 dB SPL and was gradually reduced in 5 dB steps down to 10 dB SPL.

### 2.12 Immunohistochemistry and confocal imaging

After the ABR test is completed, we used immunohistochemistry to evaluate the expression of the model genes Mef2c and Nedd4, which are implicated in ARHL, in the cochlea. We utilized 28 days-old (*n* = 3) and 12 months-old (*n* = 3) male C57BL/6J mice. Six animals were anesthetized and euthanized through cervical dislocation. Subsequently, both cochleae were promptly excised and immersed in 4% paraformaldehyde (BBI, Sangon Biotech, China) at pH 7.5, followed by overnight fixation at 4°C. Following fixation, the cochleae were subjected to decalcification for a period of 3 days in 10% ethylenediaminetetraacetic acid (EDTA, BBI, Sangon Biotech, China) in 4°C. The tissue underwent sequential dehydration steps in 10% and 20% sucrose solutions for 10 min each, followed by immersion in 30% sucrose solutions. Finally, the tissue was immersed in a mixture of 30% sucrose solution with Optimum Cutting Temperature Compound (OCT, #4583S, SAKURA, United States) for an additional 2 h. Last, it was embedded in OCT and left overnight for optimal embedding. We prepared tissue sections of 10 μm thickness using a Leica histology cryostat (CM1950, Germany). After air-drying at room temperature, the specimens were permeabilized with 1% Triton X-100 (Solarbio Life Sciences, China) for 30 min. Subsequently, blocking treatment was carried out using 5% bovine serum albumin (BSA, Sangon Biotech, China) for 1 h. Finally, the sections underwent primary antibody incubation (Mef2c Polyclonal antibody, Proteintech, 10056-1-AP or Nedd4 Polyclonal antibody, Proteintech,21698-1-AP) overnight in 4°C at a dilution of 1:200. Following three washes PBS, the samples were incubated with fluorescently labeled secondary antibodies (goat anti-rabbit for Mef2c and Nedd4, Alexa Fluor 488 # A-11034, ThermoFisher Scientific), specifically goat anti-rabbit, at room temperature for a duration of 2 h. In conclusion, the specimens underwent triple washes with PBS, were then fixed with DAPI (Sigma-Aldrich, United States), and subsequently visualized utilizing an LSM 710 confocal microscope (Zeiss, Oberkochen, Germany).

### 2.13 Statistical analysis

R software (3.6.1) and Perl software (5.30.0) were used for statistical analyses. The “limma,” “pheatmap,” “corrplot,” and “ggstatsplot” program in R was used to visualize the data. The log-rank test was used to explore significant differences. The Cox regression model was used for univariate and multivariate survival analyses, and risk variables (*p* < 0.05) from univariate analysis were chosen for multivariate analysis.

The data are expressed as the mean ± SD, and each experiment was conducted independently at least three times for accuracy. Statistical analyses were performed using Microsoft Excel and GraphPad Prism 10 software (10.3.0). One-way analysis of variance (ANOVA) was used for analysis to mean difference and independent *t*-test applied for only two groups. Two-way ANOVA was used for comparing hearing threshold. *P* value <0.05 was considered statistically significant.

## 3 Results

### 3.1 Acquisition and analysis of differentially expressed genes related to hearing loss

**FIGURE 1:**
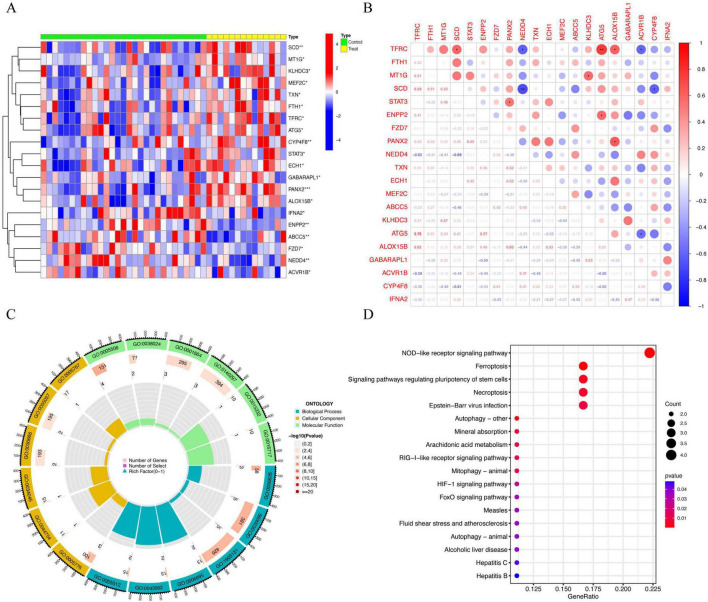
Illustrates the overview of the analysis on differentially expressed genes (DEGs) related to hearing loss. **(A)** Heatmap; the green color denotes the normal control groups, and the yellow color signifies individuals with experimental groups; **(B)** The correlation network involving 20 DEGs, with a subsequent examination of functional enrichment for genes closely associated with the DEGs. The horizontal and vertical coordinates denote differentially expressed genes, with red indicating a positive correlation and blue indicating a negative correlation. The symbol “*” in the upper right corner of the graph denotes a statistically significant correlation, and the numerical value in the lower left corner represents the specific *p*-value. The functional enrichment analysis is conducted through gene ontology (GO) in **(C)** and the Kyoto Encyclopedia of Genes and Genomes (KEGG) in **(D)**. The outermost circle depicts the GO ID, with blue denoting biological processes, yellow indicating cellular components, and green representing molecular functions. The second circle illustrates the number of genes in each GO category, while the third circle conveys the count of differentially expressed genes enriched in each GO category. The innermost circle portrays the proportion of genes involved. *, *P* < 0.05; **, *P* < 0.01.

To determine whether FRGs are differentially expressed in deaf individuals, we compared ferroptosis-related gene expression data from FerrDb database (Zhou and Bao, 2020) and lymphoblastoid cell lines originating from a family with a mutation in the 12S rRNA gene, some of whom were deaf and some of whom had normal hearing (GSE9822) (Bykhovskaya et al., 2009). We conducted a differential analysis of ferroptosis-related genes between experimental and control groups, resulting in the identification of 20 genes (*SCD, MT1G, KLHDC3, MEF2C, TXN, FTH1, TFRC, ATG5, CYP4F8, STAT3, ECH1, GABARAPL1, PANX2, ALOX15B, IFNA2, ENPP2, ABCC5, FZD7, NEDD4*, and *ACVR1B*) exhibiting statistically significant differential expression. The results of this analysis are shown in Figure 1A.

To evaluate the correlation between differentially expressed genes (DEGs), we generated correlation plots to visually represent the results. As shown in Figure 1B, the expression of SCD is positively correlated with TFRC (*p* < 0.05). PANX2 expression is positively correlated with STAT3 (*p* < 0.05). KLHDC3 expression is positively correlated with MT1G (*p* < 0.05). ATG5 expression is positively correlated with TFRC (*p* < 0.01) and ENPP2 (*p* < 0.01). ALOX15B expression is positively correlated with TFRC (*p* < 0.05) and PANX2 (*p* < 0.05). NEDD4 expression is negatively correlated with TFRC (*p* < 0.05) and SCD (*p* < 0.01), ACVR1B expression is negatively correlated with TFRC (*p* < 0.05) and ATG5 (*p* < 0.05), and CYP4F8 expression is negatively correlated with SCD (*p* < 0.05). We performed gene ontology (GO) functional enrichment analysis on DEGs and obtained statistically significant enrichment results (Figure 1C and Table 2). Additionally, we performed Kyoto Encyclopedia of Genes and Genomes (KEGG) pathway enrichment analysis on the differentially expressed genes, yielding noteworthy outcomes (Figure 1D). The gene enrichment map highlights Ferroptosis-related pathways and is associated with functions such as iron ion binding in the Molecular Function category and iron ion transport in the Biological Process category. Iron ion binding mainly describes how proteins or molecules bind to iron ions and exert their functions, while iron ion transport focuses on the movement and distribution of iron ions within the body or cells. Both are closely related to the biological function of iron and have significant implications for health.

**TABLE 2:** The functional enrichment analysis of gene ontology (GO).

Ontology	ID	Description
MF	GO:0005506	Iron ion binding
MF	GO:0001664	G protein-coupled receptor binding
MF	GO:0140297	DNA-binding transcription factor binding
MF	GO:0038024	Cargo receptor activity
MF	GO:0015232	Heme transmembrane transporter activity
MF	GO:0016717	Oxidoreductase activity, acting on paired donors, with oxidation of a pair of donors resulting in the reduction of molecular oxygen to two molecules of water
CC	GO:0005776	Autophagosome
CC	GO:0030666	Endocytic vesicle membrane
CC	GO:0055037	Recycling endosome
CC	GO:0044754	Autolysosome
CC	GO:0034045	Phagophore assembly site membrane
CC	GO:0005767	Secondary lysosome
BP	GO:0030099	Myeloid cell differentiation
BP	GO:1903131	Mononuclear cell differentiation
BP	GO:0006826	Iron ion transport
BP	GO:0006995	Cellular response to nitrogen starvation
BP	GO:0043562	Cellular response to nitrogen levels
BP	GO:0055012	Ventricular cardiac muscle cell differentiation

### 3.2 The novel model based on the lasso model and the SVM-REF model

**FIGURE 2:**
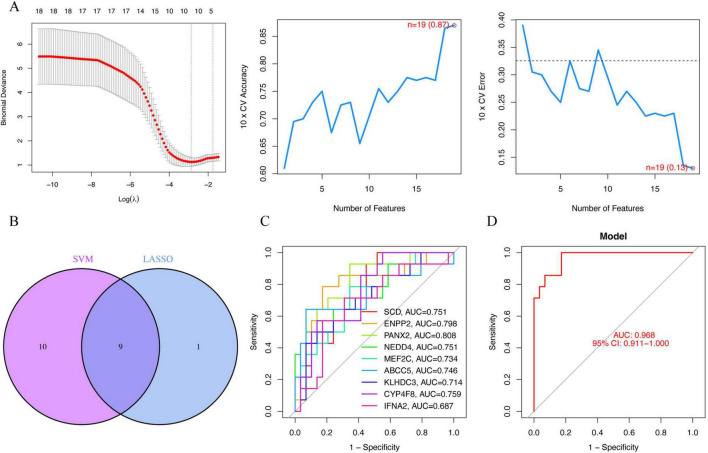
A novel model based on the Lasso model and the support vector machine recursive feature elimination algorithm (SVM-REF) model. X-axis: The top number on the horizontal axis represents the number of genes. [Log(λ)]: The horizontal axis represents the logarithmic values of λ [Log(λ)]. λ is the penalty parameter in Lasso regularization, which controls the sparsity of the model. Larger values of λ indicate stronger regularization, potentially leading to more feature coefficients being shrunk to zero. Y-axis (Binomial Deviance): The vertical axis represents the binomial deviance, which is a measure of the model’s performance in cross-validation. The lower the deviance, the better the model’s performance. **(A)** Feature maps of the Lasso regression model and SVM-REF model; **(B)** Venn plot showing the intersection genes of the lasso regression model and the SVM-REF model; The ROC curve of the novel model genes **(C,D)**.

The Lasso model was established, as illustrated in Figure 2A, where the vertical axis denotes binomial deviance. The number of feature genes corresponding to the minimum binomial deviance was determined to be 10, including *MT1G*, *SCD*, *ENPP2*, *PANX2*, *NEDD4*, *MEF2C*, *ABCC5, KLHDC3*, *CYP4F8*, and *IFNA2*. Subsequently, the SVM-REF model was constructed, revealing that the point with the highest cross-validation accuracy and the smallest error corresponded to the value of 19. This signifies that the number of SVM features is 19, specifically *CYP4F8*, *ENPP2*, *IFNA2*, *PANX2*, *ATG5*, *MEF2C*, *KLHDC3*, *STAT3*, *ECH1*, *TFRC*, *ABCC5*, *GABARAPL1*, *FTH1*, *SCD*, *NEDD4*, *ACVR1B*, *TXN*, *FZD7*, and *ALOX15B*. After taking the intersection of the characteristic genes identified by the Lasso model and the SVM-REF model, the novel model, namely *SCD*, *ENPP2*, *PANX2*, *NEDD4*, *MEF2C*, *ABCC5*, *KLHDC3*, *CYP4F8*, and *IFNA2*, was obtained, as shown in Figure 2B.

To evaluate the diagnostic accuracy of the selected feature genes for the disease, we generated a Receiver Operating Characteristic (ROC) curve, illustrated in the Figures 2C, D. The horizontal axis represents the false positive rate, while the vertical axis represents the true positive rate. The Area Under the Curve (AUC) of a ROC curve typically ranges from 0.5 to 1, with a larger area indicative of higher accuracy. The ROC curves for the nine feature genes displayed AUC values exceeding 0.7, with the exception of *IFNA2*, which fell below 0.7. This implies that each of the eight genes, excluding *IFNA2*, individually exhibits a high disease diagnosis rate. Subsequently, we amalgamated the nine genes to construct a model, yielding an AUC of 0.968, indicating an exceptionally high disease diagnosis rate.

### 3.3 Genetic immune-related analysis of the novel model

**FIGURE 3:**
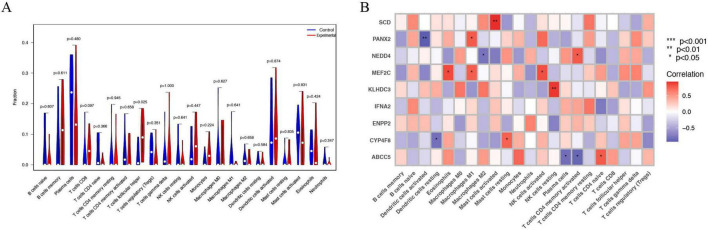
Genetic immune-related analysis of the novel model. **(A)** Differential analysis on immune cells; **(B)** Correlation analysis between feature genes and immune cells. *, *P* < 0.05; **, *P* < 0.01; ***, *P* < 0.001.

Cell-type Identification by Estimating Relative Subsets of RNA Transcripts (CIBERSORT) is a computational method for evaluating the relative abundance of different cell types in complex mixed tissue samples (Newman et al., 2015). Through the implementation of a differential analysis targeting immune cells, discernible statistical distinctions (*p* = 0.025) were observed exclusively in the expression levels of T cells follicular helper, differentiating the experimental group from the control group amid the various immune cells scrutinized. Subsequently, a correlation analysis elucidating the relationships between feature genes and immune cells was conducted, with the results being visually represented in a heatmap. Significant positive correlations (*p* < 0.01) were identified between *SCD* expression and the infiltration of activated mast cells, along with positive correlations (*p* < 0.05) between *PANX2* expression and the infiltration of M1 macrophages. Conversely, notable negative correlations (*p* < 0.01) were established between *PANX2* expression and the infiltration of activated dendritic cells. Furthermore, positive correlations (*p* < 0.05) were discerned between *NEDD4* expression and the infiltration of CD4 memory activated T cells, coupled with negative correlations (*p* < 0.05) with the infiltration of M2 macrophages. Additionally, *MEF2C* expression exhibited positive correlations (*p* < 0.05) with the infiltration of eosinophils, M1 macrophages, and activated NK cells. *KLHDC3* expression demonstrated noteworthy positive correlations (*p* < 0.01) with the infiltration of resting NK cells, while *CYP4F8* expression manifested positive correlations (*p* < 0.05) with the infiltration of resting mast cells and negative correlations (*p* < 0.05) with the infiltration of resting dendritic cells. Lastly, *ABCC5* expression revealed positive correlations (*p* < 0.05) with the infiltration of CD4 naive T cells, juxtaposed with negative correlations (*p* < 0.05) with the infiltration of plasma cells and CD4 memory activated T cells (Figure 3).

### 3.4 The novel model encompasses Gene set enrichment Analysis (GSEA) and Gene set variation Analysis (GSVA) for the investigation of genetic mechanisms

**FIGURE 4:**
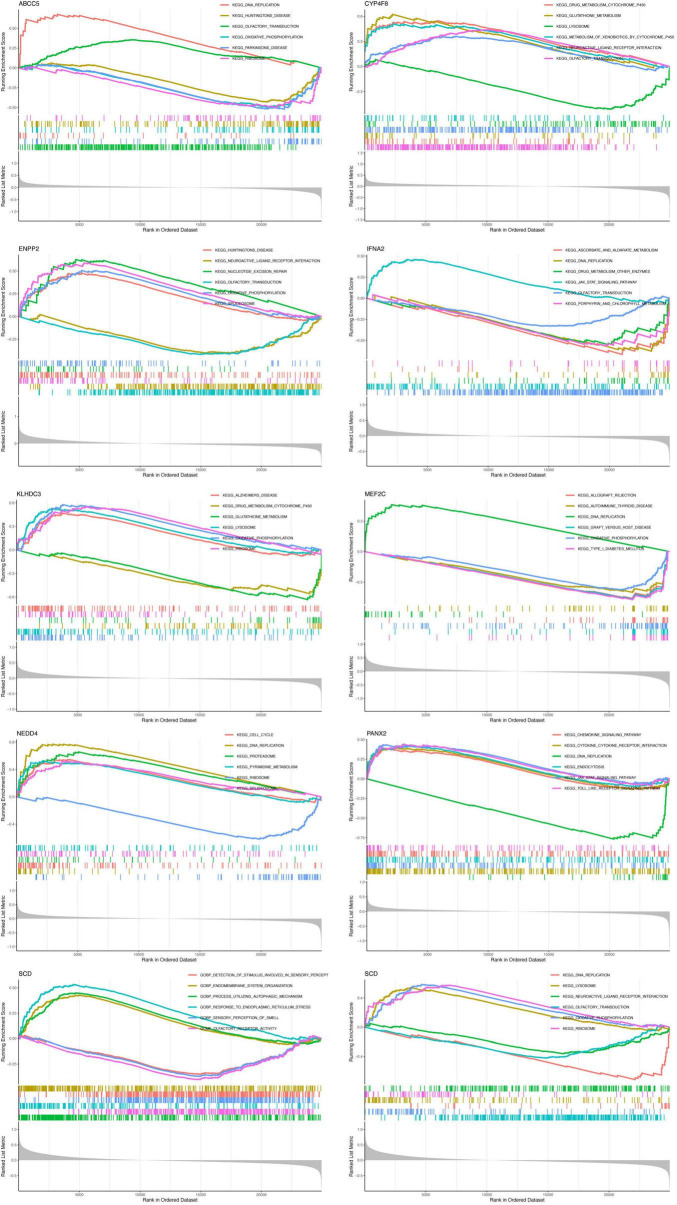
Gene Set Enrichment Analysis (GSEA) of the novel model genes. Rank in Ordered Dataset, represents the ranking value of the data set. Upper part: The enrichment curve represents the dynamic process of enrichment score (ES) scoring. The final ES value of the gene set is the peak value (highest or lowest point). A positive ES value indicates that the enrichment is at the top, that is, the enriched pathway is an up-regulated pathway; a negative ES value indicates that the enrichment is at the bottom, that is, the enriched pathway is a down-regulated pathway. Middle part: The horizontal axis represents the position of the gene. It is arranged from large to small, so the stronger the positive correlation, the higher the gene ranking position; the stronger the negative correlation, the lower the gene ranking position. The lower part is the distribution of all gene ranks after sorting. The corresponding genes in the left group are expressed in medium and high levels, and the corresponding genes in the right group are expressed in low levels. The signal-to-noise ratio (Signal2noise) corresponding to each gene is displayed in a gray area graph. Ranked list metric represents the gene ranking amount.

Gene Set Enrichment Analysis (GSEA) and GSVA (Gene Set Variation Analysis) are methods for gene set enrichment, but differ in approach. GSEA focuses on group comparisons, while GSVA analyzes gene set variation at the sample level. The source of the target genes is still the nine novel model genes. We computed the median expression value of the gene across all samples (columns), group the samples according to the expression level of the gene, and obtain high and low expression groups. Through GSEA analysis, we can observe which functions or pathways are active in the high or low expression groups of the novel model. As shown in the Figure 4, the horizontal axis represents sorted genes, and the vertical axis represents the enrichment score, with different colored curves representing different pathways or curves. If the peak of the curve appears in the upper left, it indicates that the function or pathway is active in the high expression group of the feature genes; if the peak of the curve appears in the lower right, it indicates that the function or pathway is active in the low expression group. Through GSEA analysis, we observed that *MEF2C* and *NEDD4* are significantly associated with DNA replication, which may be related to ARHL.

Through GSVA analysis, we observed the distribution of various pathways in the high and low expression groups of the novel model. In the high expression group of *MEF2C*, the following pathways were significantly associated: Asthma, Type I diabetes mellitus, Allograft rejection, Graft versus host disease and Oxidative phosphorylation, while in the low expression group, Homologous recombination, DNA replication, Sulfur metabolism and non-homologous end joining were significantly associated. The Oxidative phosphorylation, Homologous recombination, DNA replication, and non-homologous end joining associated with *MEF2C* are also closely related to aging and may be closely linked to ARHL.

**FIGURE 5:**
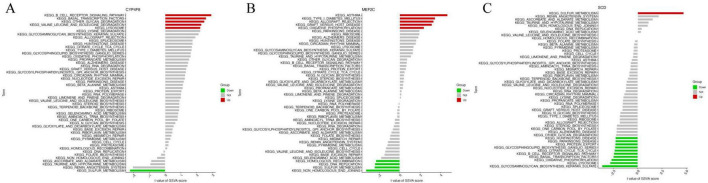
Gene Set Variation Analysis (GSVA) of the novel model genes. **(A)** GSVA of novel model gene CYP4F8. **(B)** GSVA of novel model gene MEF2C. **(C)** GSVA of novel model gene SCD. The T value of the GSVA score: the horizontal axis represents the quantified scores of the pathways related to the enrichment of the target gene across different groups (moderated t-statistic), while the vertical axis represents the signaling pathways. The target genes were divided into the high expression group (UP), low expression group (DOWN), and the group with no significant statistical differences in enriched pathways between the high and low expression groups (NOT).

In the high expression group of *CYP4F8*, the following pathways were significantly associated: B cell receptor signaling pathway, Basal transcription factors, other glycan degradation and Valine leucine and isoleucine degradation, while in the low expression group, Sulfur metabolism was significantly associated. In the high expression group of *SCD*, Sulfur metabolism was significantly associated, while in the low expression group, Huntington disease, Parkinson disease, Protein export, Glycosphingolipid biosynthesis ganglio series, Citrate cycle tca cycle, B cell receptor signaling pathway, Basal transcription factors, Oxidative phosphorylation, Lysosome and Glycosaminoglycan biosynthesis keratan sulfate were significantly associated (Figure 5 and Table 3).

**TABLE 3:** Through GSVA analysis observed the distribution of various pathways in the high and low expression groups of the novel model.

Gene	High expression groups	Low expression groups
*CYP4F8*	B cell receptor signaling pathway, Basal transcription factors, other glycan degradation, Valine leucine and isoleucine degradation	Sulfur metabolism
*MEF2C*	Asthma, type I diabetes mellitus, Allograft rejection, Graft versus host disease, Oxidative phosphorylation	Homologous recombination, DNA replication, Sulfur metabolism, non-homologous end joining
*SCD*	Sulfur metabolism	Huntingtous disease, Parkinson disease, protein export, Glycosphingolipid biosynthesis ganglio series, Citrate cycle tca cycle, B cell receptor signaling pathway, Basal transcription factors, Oxidative phosphorylation, Lysosome, Glycosaminoglycan biosynthesis keratan sulfate

### 3.5 The novel model: drug targets of genes and associated RNA prediction

**FIGURE 6:**
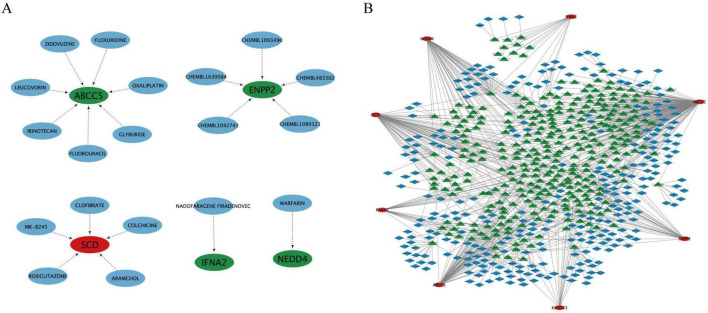
The novel model: drug targets of genes and associated RNA prediction. **(A)** The red node represents up-regulated gene, the green nodes represent down-regulated genes and the blue nodes represent related drugs. **(B)** The red ellipse represents the feature genes, the green triangle represents the miRNA associated with the feature genes, and the blue rhombus represents the lncRNA associated with the miRNA.

We used drug-gene interaction data from Dgidb (see text footnote 3) to discover potential drug targets. As shown in the Figure 6A, the red nodes represent up-regulated genes, the green nodes represent down-regulated genes, and the blue nodes represent related drugs. We found that the drugs CLOFIBRATE, COLCHICINE, ARAMCHOL, ROSIGLITAZONE, and MK-8245 can upregulate the expression of the characteristic gene *SCD*; while the drugs ZIDOVUDINE, FLOXURIDINE, OXALIPLATIN, GLYBURIDE, FLUOROURACIL, IRINOTECAN, and LEUCOVORIN can down-regulate the expression of the characteristic gene ABCC5. The drugs CHEMBL1093490, CHEMBL483302, CHEMBL1089321, CHEMBL1092743, and CHEMBL1630084 can down-regulate the expression of the characteristic gene *ENPP2*. The drug NADOFARAGENE FIRADENOVEC can down-regulate the expression of the characteristic gene *IFNA2*, and the drug WARFARIN can down-regulate the expression of the characteristic gene *NEDD4*.

We used three online tools, TargetScan (see text footnote 4), miRanda (see text footnote 5), and miRDB (see text footnote 6), to analyze the miRNAs associated with the nine novel model genes. When the corresponding database matches the relevant miRNA, the score is assigned as one. If all three databases match, the score is three. The miRNAs were matched with the corresponding lncRNA data using the spongeScan database (see text footnote 7). A miRNA-lncRNA-gene network was constructed by taking the intersection of their shared genes. As shown in the Figure 6B, red ellipse represents the feature genes (eight genes, *CYP4F8* not predicted), green triangle represents the miRNA associated with the feature genes (437), and blue rhombus represents the lncRNA associated with the miRNA (417).

### 3.6 Genetic variation of model genes (MEF2C, CYP4F8, ENPP2, NEDD4) associated with age-related hearing loss

**FIGURE 7:**
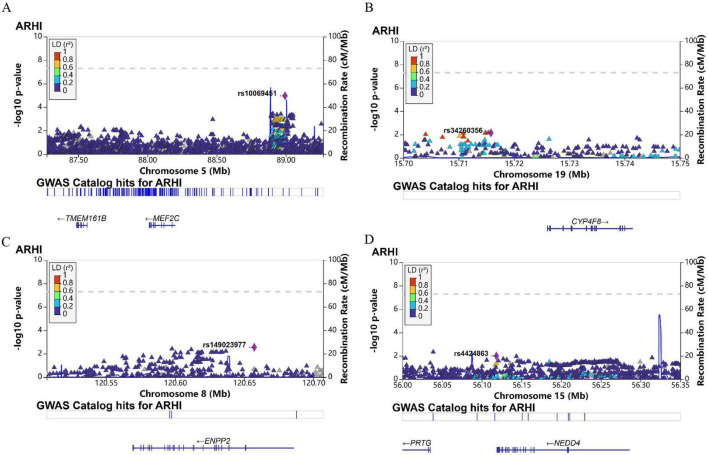
Genome-wide association studies (GWAS) analysis of age-related hearing loss (ARHL)-associated [(A) *MEF2C*, (B) *CYP4F8*, (C) *ENPP2*, (D) *NEDD4*)] single nucleotide polymorphisms (SNPs).

The result revealed that single nucleotide polymorphism (SNP) loci in four gene (*MEF2C, CYP4F8, ENPP2, NEDD4*) regions were nominally associated with ARHL. As shown in the Figure 7, the rs10069451 (C > A) located in the upstream transcriptional regulatory region of *MEF2C* shows a significant positive correlation with ARHL (*p*-value = 1.1 × 10^–5^ beta = 0.87). The rs34260356 (G > A) located in the intronic region of *CYP4F8* exhibited a suggestive association of ARHL (*p*-value = 7.3 × 10^–3^, beta = 1.05). The rs149023977 (T > C) located in the intronic region of *ENPP2* (*p*-value = 2.8 × 10^–3^, beta = 1.96) and the rs4424863 (A > T) located a non-coding SNP in the intron region of *NEDD4* (*p*-value = 0.01, beta = 0.941) also showed nominal associations.

### 3.7 Verfication ferroptosis and model genes in HEI-OC1 cells

**FIGURE 8:**
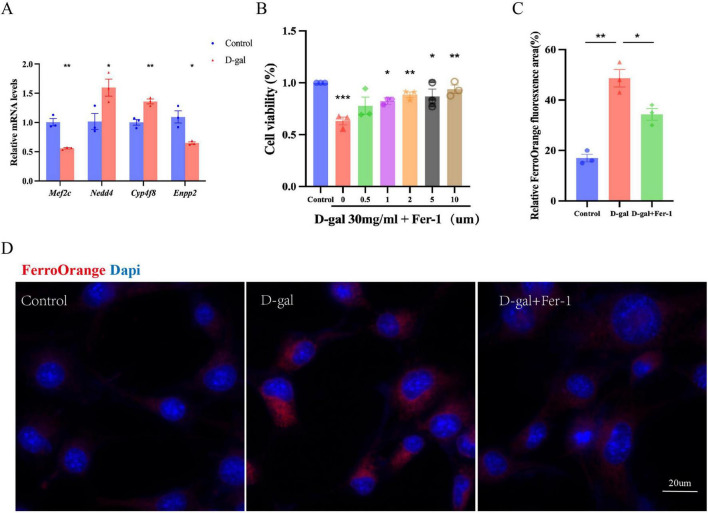
The expression of model genes associated with age-related hearing loss (ARHL) in HEI-OC1 cells. **(A)** qRT-PCR analysis (model genes *Mef2c*, *Cyp4f8, Enpp2, Nedd4*) using D-gal treated HEI-OC1 cells. **(B)** CCK-8 assay showed that Fer-1 effectively reversed the ferroptosis observed in aging HEI-OC1 cells. We performed Fe^2+^ detection to verify the occurrence of ferroptosis in aging HEI-OC1 cells, followed by fluorescence imaging **(D)** and quantitative analysis **(C)**. FerroOrange (red fluorescence) used to label Fe^2+^; DAPI (blue fluorescence) used to label the cell nuclei. Experiments were performed in triplicate, and *p*-values were determined by One-way ANOVA and *t*-test. Scale bars: 20 μm. (*, *P* < 0.05; **, *P* < 0.01; ***, *P* < 0.001).

D-gal treated HEI-OC1 cells have been utilized as an *in vitro* model to investigate the cellular and molecular mechanisms underlying ARHL. D-gal is widely used in research to simulate oxidative stress and mimic natural aging processes in both *in vitro* and *in vivo* models (John et al., 2005; Zhong et al., 2012). A total of 30 mg/ml D-gal induces a 50% cell viability in HEI-OC1 cells as reported in previous studies; therefore, 30 mg/ml D-gal was used to establish the aging model condition (He et al., 2021). In this study, we focused on the genes *Mef2c*, *Cyp4f8, Enpp2, Nedd4*, and conducted qRT-PCR analysis using D-gal treated HEI-OC1 cells. As depicted in Figure 8A, we observed downregulation of *Mef2c* (*p* < 0.01) and *Enpp2* (*p* < 0.05) expression in D-gal treated HEI-OC1 cells compared to the control group. Conversely, the expression levels of *Cyp4f8* (*p* < 0.05) and *Nedd4* (*p* < 0.01) were significantly up-regulated in the D-gal treated group compared to the control group. These findings suggest that the expression of certain model genes (*Mef2c*, *Cyp4f8, Enpp2, Nedd4*) is modulated by D-gal treatment.

To verify the occurrence of ferroptosis in aging HEI-OC1 cells, we performed Fe^2+^ detection. FerroOrange is a fluorescent probe was used to detect unstable divalent Fe^2+^, emitting irreversible orange fluorescence upon interaction with Fe^2+^. The results showed that after treatment with 30 mg/ml D-gal for 48 h, the expression of Fe^2+^was increased (*p* < 0.001; Figures 8C, D). To better demonstrate the occurrence of ferroptosis in aging HEI-OC1 cells, we used CCK-8 assays and found that 1 μM of Fer-1 (a ferroptosis inhibitor) could mitigate D-gal-induced cell damage (*p* < 0.05; Figures 8C, D). Moreover Fer-1 (1 μM) effectively reversed the ferroptosis observed in aging HEI-OC1 cells (*p* < 0.05 Figure 8B).

### 3.8 The expression of model genes in cochlea

**FIGURE 9:**
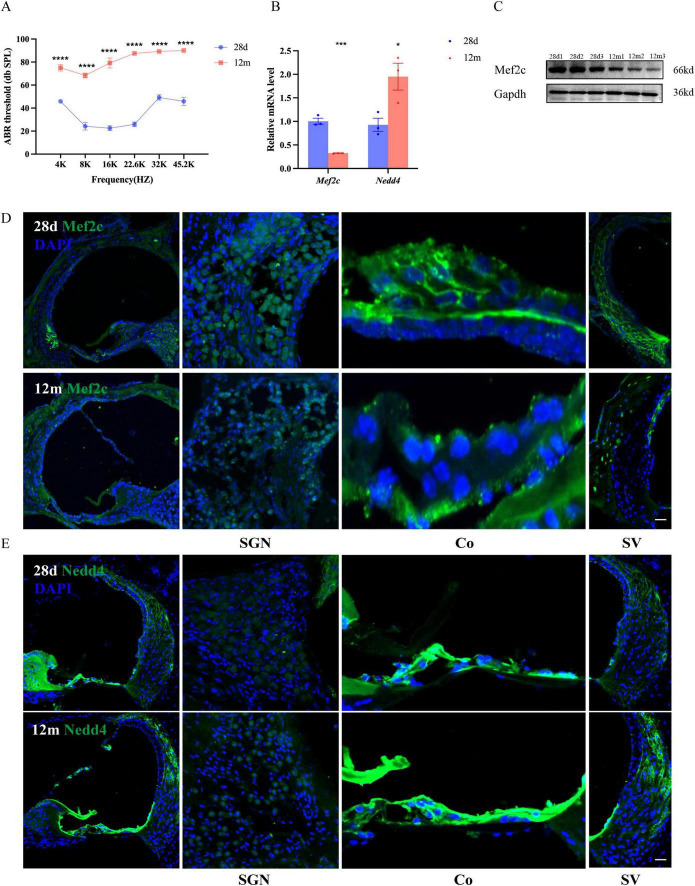
Expression of Mef2c and Nedd4 in the cochlea. **(A)** Auditory brainstem response (ABR) thresholds measured at 28 days of age in C57BL/6J mice (*n* = 6 per group). Data are presented as mean ± SEM and were computed from tone burst responses at frequencies of 4, 8, 16, 22.6, 32, and 45.2 kHz. **(B)** RNA expression levels in the cochleae of 28 days-old C57BL/6J mice (*n* = 3 per group). **(C)** Protein expression levels in the cochleae of 28 days-old C57BL/6J mice (*n* = 3 per group). **(D,E)** Immunofluorescence analysis of Mef2c (C, green fluorescence) and Nedd4 (D, green fluorescence) was performed in the organ of Corti (Co), spiral ganglion neurons (SGNs), and stria vascularis (SV) of 28 days-old and 12 months-old mice, with DAPI (blue fluorescence) used to label the cell nuclei. Experiments were performed in triplicate, and *p*-values were determined by *t*-test and Two-way ANOVA. Scale bars: 20 μm. (*, *P* < 0.05; ***, *P* < 0.001; ****, *P* < 0.0001).

After performing auditory brainstem response (ABR) testing, we found that the hearing of 12 months-old C57 mice significantly declined across all frequency ranges compared to 28 days-old mice (Figure 9A, *P* < 0.001), which is consistent with previous study (Sun et al., 2023). Then, we utilized qRT-PCR to further verify the expression of model genes in the cochleae of 28 days-old and 12 months-old mice. As shown in Figure 9B, the mRNA expression of *Mef2c* in the cochleae of 12 months-old mice was significantly lower than in 28 days-old mice (*p* < 0.01), while the expression of *Nedd4* was significantly higher (*p* < 0.05). The Western blot experiment further confirmed that the expression level of MEF2C in the cochlear tissue of 12 months-old mice was significantly lower than 28 days-old mice (Figure 9C).

To verify expression patterns of Mef2c and Nedd4 in the cochlea, immunohistochemistry was conducted. Our analysis revealed significant Mef2c expression in spiral ganglion neurons (SGNs) and bone-forming cells surrounding the cochlear lateral wall. Notably, Mef2c expression levels were notably higher in younger mice, particularly at postnatal stages, compared to 12 months-old mice. Additionally, Nedd4, a known cochlear marker, exhibited robust expression in the organ of Corti (Co), Reissner’s membrane, SGNs, and stria vascularis (SV). Furthermore, Nedd4 expression levels were observed to be higher in the cochleae of 12 months-old mice compared to those at 28 days of age (Figures 9D, E and Supplementary Figure 1).

## 4 Discussion

Hearing loss represents a global health challenge with complex etiologies, where programmed cell death pathways, including ferroptosis, have emerged as critical contributors. Our study integrates multi-omics approaches to systematically identify ferroptosis-related genes (FRGs) associated with hearing loss and establishes their diagnostic and therapeutic potential. The intersection of bioinformatics predictions, GWAS validation, and experimental evidence highlights the following key insights.

Our identification of 20 FRGs differentially expressed in hearing loss underscores the involvement of ferroptosis in cochlear pathophysiology. Notably, the nine-gene signature (*SCD*, *ENPP2*, *PANX2*, *NEDD4*, *MEF2C*, *ABCC5*, *KLHDC3*, *CYP4F8*, *IFNA2*) encompasses regulators of iron metabolism (e.g., *SCD*), lipid peroxidation (*ENPP2*), and redox balance (*KLHDC3*), aligning with the hallmarks of ferroptosis (Dixon et al., 2012; Tang et al., 2021; Zhang et al., 2022; Sen et al., 2023) (Bai et al., 2018). Functional enrichment analysis further links these genes to iron ion transport and autophagosome pathways, suggesting that iron embolism imbalance and impaired autophagy may synergistically drive cochlear cell death. Bioinformatics functional analysis indicated that nine Ferroptosis-Related Genes (FRGs) were predominantly enriched in Nucleotide-binding Oligomerization Domain (NOD) and ferroptosis pathways. The NOD pathway is an important cellular signaling pathway primarily associated with the inflammatory responses and programmed cell death. By promoting the release of cytokines such as IL-1β, IL-6, and TNF-α, and regulate the processes of programmed cell death, the NOD pathway can lead to hearing loss (Frye et al., 2019). Moreover, we hypothesized that ferroptosis played an important role in the development of hearing loss. ROC curve analysis also revealed that 9 FRGs might be accurately distinguished from the normal samples. Finally, we further explored whether risk genes are involved in ARHL in human. Interestingly, through genetic analysis, it has been determined that variant sites in four genes are associated with ARHL, suggesting potential regulatory functions. Previous studies have confirmed the occurrence of ferroptosis-related mechanisms in the auditory cortex of D-gal induced aging rats (Chen et al., 2020). Moreover, oxidative stress levels and lipid peroxidation markers in the cochlea of aged mice are significantly elevated also leading to ferroptosis (Sun et al., 2023). These findings extend prior work on ferroptosis in other diseases to auditory pathologies.

The association of MEF2C and NEDD4 with ARHL through GWAS and cochlear validation provides mechanistic insights into age-dependent auditory decline. MEF2C, a transcription factor critical for neuronal survival (Harrington et al., 2016; Santos et al., 2017), may safeguard cochlear neurons by suppressing ferroptosis, as its silencing exacerbates Erastin-induced cell death (Bao et al., 2021). *MEF2C* deficiency leads to a mild decrease in auditory sensitivity in young adult mice, moreover it is also identified as a potential crucial gene in the development of the AN (McChesney et al., 2022). Similarly, NEDD4, an E3 ubiquitin ligase, could modulate auditory function by regulating ion channel turnover or stress-response proteins in the stria vascularis (Lewis et al., 2018). The ARHL-linked SNPs in MEF2C (rs10069451) and NEDD4 (rs4424863) localize to regulatory regions, potentially altering their expression or splicing in aging cochleae. Expressed broadly within the cochlear duct, *NEDD4* encodes a ubiquitin ligase protein known for its interaction with and ubiquitination of products from diverse genes associated with deafness (Zhong and Liu, 2009). Therefore, *NEDD4* may serve as novel candidate deafness gene. This aligns with reports of elevated oxidative stress and lipid peroxidation in aged murine cochleae (Sun et al., 2023), suggesting ferroptosis as a unifying mechanism across genetic and age-related hearing loss.

Our drug-gene interaction network identifies candidates (e.g., CLOFIBRATE for SCD upregulation, WARFARIN for NEDD4 inhibition) that warrant validation in preclinical models. Notably, SCD overexpression has been shown to mitigate ferroptosis in hepatocytes (Bai et al., 2018), supporting its therapeutic potential for hearing preservation. Furthermore, immune infiltration analysis reveals significant correlations between FRGs (e.g., MEF2C with NK cells, PANX2 with M1 macrophages) and pro-inflammatory subsets, suggesting that ferroptosis may amplify cochlear inflammation—a known driver of sensorineural hearing loss (Zhang et al., 2020; Grayson et al., 2022). Immune cells play a significant role in the development and progression of hearing loss, especially in conditions like sensorineural hearing loss, otitis media, and age-related hearing decline. Targeting these interactions could offer dual benefits by reducing both cell death and immune-mediated damage. Moreover, the immune system’s response can influence the inner ear’s health, sometimes contributing to tissue damage and hearing loss.

However, while our study provides a robust framework, certain limitations must be acknowledged. First, the reliance on lymphoblastoid cell line data (GSE9822) introduces potential bias, as peripheral blood cells may not fully recapitulate cochlear gene expression. It is important to note that mitochondrial 12S rRNA mutations have been associated with hearing loss in multiple families, underscoring their biological significance (Chen and Guan, 2022). To address this issue and enhance the robustness of our research, we plan to incorporate more database validation in our future work, such as data derived from mouse models, which may provide further insights into the implications of these mutations. Future studies should validate FRG expression in human temporal bone specimens or murine cochlear explants. In our upcoming in-depth research, we plan to perform single-cell RNA sequencing using human temporal bone specimens or cochlear-specific datasets, and will strive to expand the scope of data inclusion as much as possible. In this study, we utilized HEI-OC1 cells and C57BL/6J mice, which are commonly adopted models in auditory research. However, the C57BL/6J strain carries a *Cdh23* mutation, which imposes inherent limitations as a model for ARHL. Moreover, in subsequent research, primary cell models or organoids could enhance translational relevance, and validation in alternative strains or human-derived models would provide greater precision. While our study is a candidate gene investigation focusing on MEF2C, CYP4F8, ENPP2, and NEDD4, there is prior biological evidence linking these genes such as NEDD4 and MEF2C to auditory function and ARHL (Lewis et al., 2018; McChesney et al., 2022). Therefore, we choose a relaxed significance threshold in GWAS. In the future research, we will perform overexpression or knockout validation of key genes in the upcoming experiments to further confirm their role in ARHL and conduct functional validation through immune cell infiltration analysis to confirm their direct contribution to cochlear damage.

## 5 Conclusion

Ferroptosis may be a potential process in the occurrence and development of hearing loss. A comprehensive bioinformatics analysis was conducted utilizing diverse datasets to explore the expression profiles and diagnostic significance of *SCD, MEF2C, NEDD4, PANX2, ENPP2, KLHDC3, CYP4F8, ABCC5* and *IFNA2* in patients with hearing loss. These genes have the potential to be diagnostic biomarkers for hearing loss. Moreover, variant sites in *MEF2C* and *NEDD4* suggest potential regulatory functions in ARHL, offering novel targets for interventions aimed at preserving auditory function in aging populations. Future work should prioritize *in vivo* validation of these candidates and explore combinatorial strategies targeting both ferroptosis pathways.

## Data Availability

The datasets presented in this study can be found in online repositories. The names of the repository/repositories and accession number(s) can be found below: https://www.ncbi.nlm.nih.gov/geo/, GSE9822.

## Footnotes

1
https://www.ncbi.nlm.nih.gov/geo/

2
http://zhounan.org/ferrdb/current/

3
https://www.dgidb.org/

4
http://targetscan.org

5
https://regendbase.org/tools/miranda

6
http://www.mirdb.org/

7
http://spongescan.rc.ufl.edu/

8
https://www.ebi.ac.uk/
