# Supplementary figures and images for: Experimental validation and identification of ferroptosis-associated biomarkers for diagnostic and therapeutic targeting in hearing loss
Source: Front Aging Neurosci. 2025 Apr 25;17:1526519. doi: 10.3389/fnagi.2025.1526519 (PMC12062182; doi:10.3389/fnagi.2025.1526519)

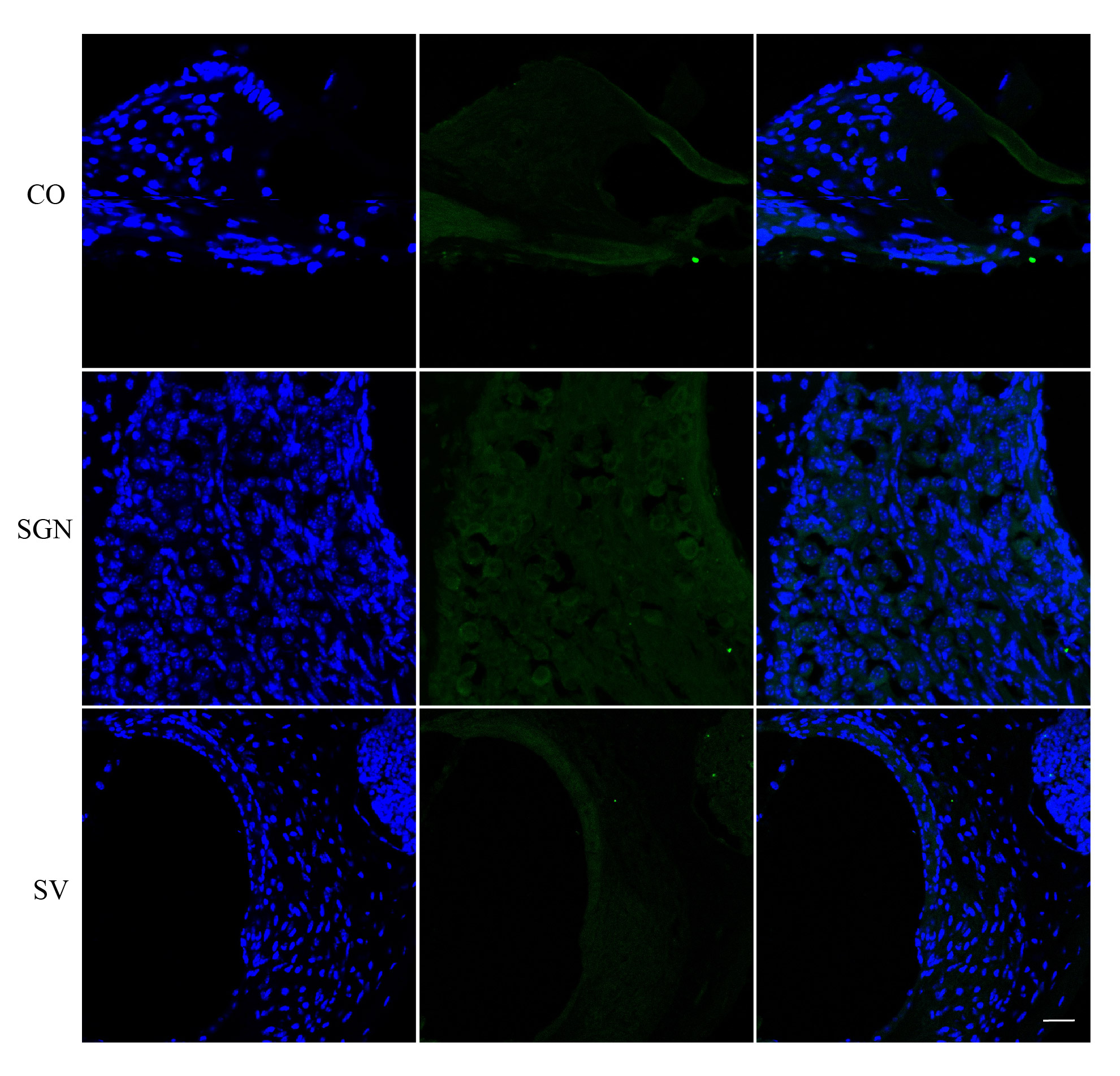

Supplement: Supplementary file 1 [file Image_1.jpeg]
